# Assessment of Vitamin A Status of Preschool Children in a Sub-Saharan African Setting: Comparative Advantage of Modified Relative-dose Response Test

**DOI:** 10.3329/jhpn.v28i5.6157

**Published:** 2010-10

**Authors:** C. Samba, B. Gourmel, P. Houze, D. Malvy

**Affiliations:** ^1^ Department of Tropical Medicine, Hôpital Saint-André, University Hospital Center of Bordeaux and Centre René Labusquière (Tropical Diseases Branch), University Victor Segalen Bordeaux 2, F-33076 Bordeaux, France; ^1^ Centre d'Etudes et de Recherche Médecins d'Afrique, Brazzaville, Congo and Médecins d'Afrique Brazzaville, Park 172, rue des fleurs. Quartier Ravin du Tchad, Brazzaville, République du Congo; ^1^ UNICEF Brazzaville (Congo), BP 2110, D-34 Rue Lucien Fourneau, Brazzaville, République du Congo; ^2^ Laboratoire de Biochimie A, Hôpital Saint Louis, 1 Avenue Claude Vellefaux, F-75010 Paris, France

**Keywords:** Vitamin A deficiency, Xerophthalmia, Congo

## Abstract

A nationally-representative sample of 2,696 preschool children living in Congo was examined during August-September 2003 to determine the rates of vitamin A deficiency. Ninety clusters of 30 children, aged six months to six years, were selected, using a randomized two-level cluster-sampling method. Vitamin A deficiency was determined by assessing the prevalence of active xerophthalmia (nightblindness and/or Bitot spots) in the cross-over sample of 2,696 individuals. A semi-quantitative seven-day dietary questionnaire was concurrently applied to the mothers of children enrolled to estimate the latter's consumption of vitamin A-rich food. Vitamin A status was assessed by performing the modified relative dose-response test (MRDR) on dried blood spots (DBS) from a subsample of 207 children aged less than six years and the impression cytology with transfer (ICT) test on a subsample of 1,162 children. Of the children enrolled, 5.2% suffered from nightblindness, 8.0% had Bitot spots, and 2.5% had other vitamin A deficiency sequellae. Fifty-three percent of the ICT tests showed the presence of vitamin A deficiency. The biochemical MRDR test showed that the vitamin A status of 30% of the study children was critical. Twenty-seven of them had retinol levels of <10 μg/dL [mean±standard deviation (SD) 7.02±2.0 μg/dL], and 50% had retinol levels of 10-20 μg/dL (mean±SD 14.2±2.83 μg/dL). The poor health status and low rates of consumption of vitamin A-rich food are the main factors determining critical status. Vitamin A deficiency, reflecting poor nutrition and health, is a serious public-health issue among children aged less than six years in Congo.

## INTRODUCTION

Vitamin A deficiency that causes xerophthalmia is endemic in sub-Saharan African countries with few resources. When the logistic conditions make it difficult to obtain serum samples for measuring retinol levels, the dried blood spot (DBS) technique is a useful tool for field studies on vitamin A status (1). We, therefore, developed a suitably-sensitive modified relative dose-response method (MRDR) on DBS (1) for determining the parameters of vitamin A status in groups of children with a risk of deficiency. We subsequently carried out assessments of prevalence of vitamin A deficiency, using the serum retinol concentrations analyzed in DBS on children in the Republic of Congo (2). The aim of the present survey was to improve the present picture of xerophthalmia using both MRDR on DBS and ICT test and describing other determinants of vitamin A status in children in the setting of this equatorial African country.

## MATERIALS AND METHODS

### Study areas and setting

**Fig. 1.**
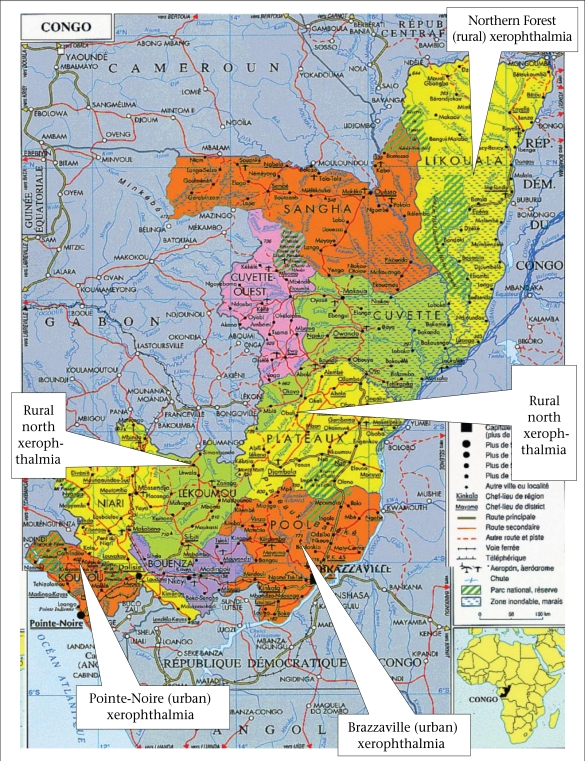
Distribution of xerophthalmia in the Republic of Congo

This study was carried out at the end of the dry season—from August to September 2003—in five regions (in Brazzaville and the town of Kouilou ‘Pointe-Noire’, which are referred to here as urban settings, and in the Lekoumou, the Likouala, and the plateau, which are collectively referred to as ‘the rural north’) (Fig. 1). This study period was chosen because vitamin A status was assumed to be the lowest level at that time of the year. The regions studied were selected using the following criteria: accessibility of the villages, cooperation of village leaders, and socioeconomic and ecological conditions. The diet of these populations consists mainly of dark green-leafy vegetables and cassava tuber (the staple food), sweet potatoes, and bananas and has a relatively-poor energy content. The protein intake is known to be dramatically low in these regions at the start of the rainy season, which lasts from October to December (3).

### Sampling methods

The population sample studied was recruited using a two-level, cross-sectional random cluster sample design, based on the procedures recommended by the Blindness Prevention Programme of the World Health Organization (WHO) (4). The study was planned using a two-stage cluster-sampling method, with stratification depending on agro-ecological factors. For this purpose, we used information provided by the first national nutrition and health survey which was conducted on all eligible households, whatever be the nationality of their occupants and the length of time for which they had been living in the area (5). The target sample of 2,700 children, aged six months to six years, was assessed, based on an expected prevalence of vitamin A deficiency of 4%, an alpha error of 5%, and an absolute accuracy requirement of 1%. The criterion defining vitamin A deficiency was the presence of active xerophthalmia [Bitot spots (X_1_B], active corneal disease (X_2_) and/or nightblindness (XN).

**Fig. 2.**
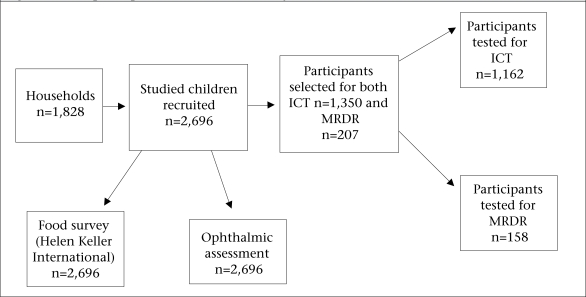
Flow of participants enrolled in the study

Thirty children from each of the 90 villages (clusters) were selected at random, amounting to a targeted total of 2,700 individuals in all. If a cluster was too small to be able to recruit 30 children, the nearest village was used for completing the cluster. Cluster effects were not taken into consideration in determining the sample-size. Three sets of areas were selected: the rural north area (30 clusters), Brazzaville (30 clusters), and Kouilou (30 clusters). Several subsamples were selected at random. One in every two children was enrolled for the assessment of vitamin A status by performing the conjunctival transfer ICT test (expected sample-size n=1,350), and one in every 13 children for assessment of biochemical status using the MRDR test (expected sample-size: n=207). A flow-chart showing the participants in each assessment group is given in Figure 2.

ICT=Impression cytology with transfer; MRDR=Modified relative-dose response

### Measurements and indicators

Data relating to socioeconomic status, morbidity events, clinical ophthalmologic examination, and histological test and biochemical data on each child were collected based on DBS tests. In addition, dietary details were collected using a ready-made pre-tested questionnaire containing specific food items that were carefully selected and tested using the food-frequency method described in a previous paper (6).

### Morbidity

Information on morbidity was collected by interviewing the mothers. A two-week history of morbidity symptoms, including any episodes of fever and/or diarrhoea was noted. A six-week history of measles, any immunization exposure, and any recalled additional use of vitamin A capsules during the previous three months were also noted. Children were subdivided into three groups: fever, measles, and diarrhoea. In patients with a history of measles, the date of its occurrence was recorded. Those suffering from diarrhoea had to meet the following criteria: loss of weight of over 10% and number of liquid stool episodes per day greater than 4.

### Vitamin A intake

*Food survey*: Information on breastfeeding and age at weaning was obtained by interviewing the mothers. Dietary information was based on a questionnaire with specific items selected and used by the HKI food frequency method (Helen Keller International, 1994) (6).

These food items included:

A staple food that is consumed by most children on a daily basis—the first food-frequency question asked focused on the most commonly-consumed staple food, to put the respondent at ease by eliciting a positive (e.g. ‘seven days a week’) response.
A food that is almost never consumed by young children in the survey area—this food-frequency question focused on a spicy food item (hot pepper) which will elicit a negative response in most communities.
Main sources of vitamin A—these food items contain at least 100 retinol equivalents (REs) of vitamin A per 100 g of the food.
Main sources of fat, oil, and protein—adequate consumption of these items is necessary for the absorption and peripheral use of vitamin A by humans.

A ready-made pre-tested seven-day food-frequency questionnaire was administered to the respondents to determine how often food containing vitamin A and carotenoids had been consumed during the previous weeks. This information was collected on all the 2,696 participants. Trained local health practitioners compiled a list of available foods, which were classified in four categories: (a) animal products (fresh and dried fish, eggs, and milk); (b) dark green-leafy vegetables (cassava leaves and wild green leaves); (c) fruit; and (d) palm oil. In each category, the children (n=2,696) were classified as frequent consumers, i.e. once or more per week (≥1 x/week) and infrequent consumers, i.e. less than once a week (<1 x/week).

### Ophthalmologic assessment

Trained ophtalmologists performed ophthalmic examination using an X 2.5 loupe, along with an electric torch. Any history of nightblindness (XN stage) was obtained by interviewing the mothers of children using a vernacular term. Xerophthalmia was defined as the occurrence of Bitot spots, specific active corneal lesions, or nightblindness. The children were concurrently screened for blindness trachoma, using the simplified trachoma grading system of the WHO based on the stages in the condition: inflammation plus follicular trachoma or inflammation plus intense trachoma—the presence of at least one of these stages in either eye being taken to define active trachoma (7). Vitamin A treatment (one 200,000 IU X 3 capsules) was given to all the children showing clinical signs of vitamin A deficiency, and tetracycline ointment was applied to cases with ocular infection.

### Vitamin A status

#### Impression cytology with transfer

Of the 1,350 individuals on whom it was planned to perform ICT, the test was actually performed on a sample of only 1,162 individuals because some children refused to cooperate. It was performed on both eyes of each individual as previously described (8). Four levels of classification (normal ‘N’, marginal plus ‘M+’, marginal minus ‘M-’, and deficient ‘D’) were defined, depending on the presence (normal status) or absence (deficient status) of goblet cells and on the morphology of the epithelial cells. Findings of impression cytology corresponding to deficient status were defined as critical peripheral availability corresponding to a liver vitamin A concentration of <20 μg/g.

#### Biochemical markers

Vitamin A status was assessed by applying the MRDR test to DBS (1). On the morning of the study, each child was given a dose of 8.8 μmol (2.5 mg) of 3, 4-didehydroretinol administered in acetate ester form (DR-acetate) dissolved in 250 μL of corn-oil and usually at home (9).

Later in the morning, four hours after administering DR-acetate, capillary blood was extracted by pricking the child's finger with a vaccination scratch-blade, and around 0.5 mL of blood was collected on a ‘Schleicher and Schuel, Keene, NH-USA 03431’ filter paper. The DBS were placed in plastic ziplock bags and kept away from daylight. These spots were frozen and stored until they were transferred for analysis. The conditions of transport, storage, and conservation were identical to those described by Craft *et al*. (10). Just before the assay was performed, the samples were thawed slowly to room temperature with minimum exposure to light. The serum was extracted with ethanol/hexane. We have developed a high-performance liquid chromatography (HPLC) method as previously described, with electrochemical detection (measurement potential+0.80 V) for measuring both retinol (R) and 3, 4-didehydroretinol (DR) in DBS (1). Retinol acetate was used as the internal standard. The method is linear up to 2.5 μM, with a detection limit of 0.04 μM. It is accurate to within 10%. The DBS retinol and DR recovery rates are approximately 90% (1). A molar DR/R ratio was calculated. Children with DR/R values of ≥0.06 were taken to belong to the critical vitamin A status group (9). DBS retinol concentrations were classified according to the WHO criteria for determining whether vitamin A deficiency is a public-health issue (4). Retinol values of <10 μg/dL, those between 10 and 20 μg/dL, and those of ≥20 μg/dL were defined as deficient, low and normal status respectively.

### Statistical analysis

Statistical comparisons were carried out using the chi-square test for categorical variables. Continuous data across groups were compared by performing analyses of variance. Data were analyzed using the Epi Info software (version 6.04b).

### Ethics

The procedure used was approved by the ethical committees of both French and Congolese Ministries of Health and a consent form written in French and translated into the local language was signed by the mothers of infants before examination.

## RESULTS

### Population characteristics in various regions

The survey sample of 2,696 children, aged 6-71 months, were recruited from among a population of 1,828 households in the areas studied (Table 1). Overall, boys accounted for 51% and girls for 49% of the sample.

**Table 1.** Characteristics of study population (n=2,696)

Children aged 6 months to 6 years	Brazzaville (urban) No. (%), 95% CI	Kouilou (urban) No. (%), 95% CI	Rural north No. (%), 95% CI	All samples No. (%), 95% CI
Households	658	721	449	1,828
Boys	483/928 (52.0), 48.7-55.3	463/912 (51.0), 47.7-54.3	425/856 (49.6), 46.2-53.0	1,371/2,696 (51.0), 49.1-52.9
Girls	445/928 (48.0), 44.7-51.3	449/912 (49.0), 45.7-52.3	431/856 (50.4), 47.0-53.8	1,325/2,696 (49.0), 47.1-50.9

CI=Confidence interval

### Morbidity and socioeconomic status

In the five areas studied, 28% of the patients were suffering from fever of various origins. Overall, 8.5% of the children had suffered from measles, and 16% of them had suffered from diarrhoea during the previous two weeks (Table 2). In Brazzaville, 25% (n=231) of the individuals had suffered from fever compared to 31% and 27% in Kouilou (n=284) and the rural north (n=230) respectively. In Brazzaville, 11% (n=101) of the subjects had suffered from measles compared to 7% (n=60) and 8% (n=67) in Kouilou and the rural north respectively. In Brazzaville, 16% (n=151) of the subjects had suffered from diarrhoea compared to 15% (n=144) and 16% (n=135) in Kouilou and the rural north respectively.

**Table 2.** Prevalence of infectious diseases among Congolese preschool children

Morbidity	Brazzaville (urban) No. (%), 95% CI	Kouilou (urban) No. (%), 95% CI	Rural north No. (%), 95% CI	All samples No. (%), 95% CI
Various types of fever	231/928 (25.0), 22.0-28.0	284/912 (31.0), 28.0-34.0	230/856 (27.0), 24.0-30.0	745/2,696 (28.0), 26.0-30.0
Measles	101/928 (11.0), 9.0-13.0	60/912 (7.0), 6.0-8.0	67/856 (8.0), 6.0-10.0	228/2,696 (8.5), 7.5-9.5
Diarrhoea	151/928 (16.0), 14.0-19.0	144/912 (15.0), 13.6-16.4	135/856 (16.0), 13.0-18.0	430/2,696 (16.0), 14.0-17.0

CI=Confidence interval

The socioeconomic status of the populations (living on small plots of land, access to drinking-water) in the study areas was mainly consistent with poor living conditions (Table 3). Overall, 72% and 66% of the households were living on small plots of land and had no access to drinking-water respectively.

**Table 3.** Vitamin A status and intake among Congolese preschool children in various regions

Parameter	Brazzaville (urban) No. (%), 95% CI	Kouilou (urban) No. (%), 95% CI	Rural north No. (%), 95% CI	All samples No. (%), 95% CI	p value
ICT deficiencies	306/444 (69.0), 67.2-71.0	163/387 (42.0), 40.0-44.0	148/331 (45.0), 43.0-47.0	617/1,162 (53.0), 51.0-55.0	<0.001
MRDR ≥0.06	34/83 (41.0), 29.0-50.0	13/75 (17.0), 7.0-23.0	ND	47/158 (30.0), 21.0-35.0	<0.001
Retinol levels (μg/dL)					
<10	24/83 (29.0), 7.4±1.9	19/75 (25.0), 7.2±1.5	13/49 (26.5), 6.45±2.35	56/207 (27.0), 7.02±2.0	<0.001
10-20	39/83 (47.0), 14.05±2.85	42/75 (56.0), 14.0±3.0	23/49 (47.0), 14.55±2.65	104/207 (50.0), 14.2±2.83	<0.001
XN	15/928 (2.0), 1.0-3.0	45/912 (5.0), 3.5-6.0	71/856 (8.0), 6.0-10.0	140/2,696 (5.2), 4.3-6.1	<0.001
X_1_B	56/928 (6.0), 4.5-7.7	17/912 (2.0), 0.8-3.0	142/856 (17.0), 14.0-19.0	215/2,696 (8.0), 7.0-9.0	<0.001
X_2_, X_3_A	23/928 (2.5),1.5-3.5	ND	21/856 (2.5), 1.5-3.5	44/1,784 (2.5), 1.8-3.3	<0.001
Trachoma	495/928 (53.0), 50.0-57.0	16/912 (1.8), 0.9-2.7	825/856 (96.5), 95.0-98.0	1,336/2,696 (50.0), 48.0-52.0	<0.001
NFC <once a week (%)					
Food of animal origin	(476/928) (51.0), 48.0-54.0	516/912 (56.5), 53.0-59.0	659/856 (77.0), 74.0-80.0	1,651/2,696 (61.0), 59.0- 63.0	
Vegetables	(577/928) (62.0), 59.0-65.0	623/912 (68.0), 65.0-71.0	631/856 (74.0), 71.0-77.0	1,831/2,696 (68.0), 66.0-70.0	
Fruits	(593/928) (64.0), 61.0-67.0	705/912 (77.0), 74.0-80.0	643/856 (75.0), 72.0-78.0	1,941/2,696 (72.0), 70.3-73.7	
Fat and oil	(386/928) (41.5), 38.0-44.7	357/912 (39.0), 36.0-42.0	499/856 (58.0), 54.6-61.4	1,242/2,696 (46.0), 44.0- 48.0	
MFI (days/week)	5	4.8	2.7	4.17	
Weighted total	4.9	4.4	3.9	4.4	
All items					
AN and VG	508/928 (55.0), 52.0-58.0	550/912 (60.0), 57.0-63.0	608/856 (71.0), 68.0-74.0	1,666/2,696 (62.0), 60.0-64.0	
PL	655/928 (71.0), 68.0-74.0	848/912 (93.0), 91.0-95.0	426/856 (50.0), 47.0-53.4	1,929/2,696 (72.0), 70.0-74.0	
No ADW	697/928 (75.0), 73.0-77.0	713/912 (78.0), 76.0.0-80.0	469/856 (55.0), 53.0-57.0	1,779/2,696 (66.0), 64.0-68.0	
VAS	711/928 (77.0), 74.0-80.0	309/912 (33.0), 30.0-36.0	673/856 (79.0), 76.0-81.0	1,688/2,696 (63.0), 61.0- 64.0	<0.05

AN=Food of animal origin;

CI=Confidence interval;

ICT=Impression cytology with transfer;

MFI=Mean frequency of intake;

MRDR=Modified relativedose response;

ND=Not defined;

NFC=Infrequent consumer;

No ADW=No access to drinking-water;

PL=Plot of land;

%=Proportion;

XN=Nightblindness;

X_1_B=Bitot spots;

X_2_, X_3_A=Active xerophthalmia;

VAS=Vitamin A supplements;

VG=Vegetables

The rate of vitamin A supplementation coverage was 77% in Brazzaville and 33% in Kouilou compared to 79% in the rural north area, based on the fact that the rate of national vitamin A supplementation coverage was estimated to be 63% (Table 3).

### Vitamin A intake

Table 3 shows the dietary habits of the 2,696 children enrolled and their weekly intake rates of food containing vitamin A.

As far as the dietary energy intake was concerned, infrequent consumers of vegetable-oil accounted for 41.5% in Brazzaville, 39% in Kouilou, and 58% in the rural north.

More than 77% of the study children in the rural north had not consumed any vitamin A-rich food of animal origin during the previous week compared to 51% and 56.5% in the Brazzaville and Kouilou areas respectively

The infrequent consumers of vegetables accounted for 62.0% in Brazzaville, 68% in Kouilou, and 74% in the rural north.

Fifty-five percent infrequently consumed vitamin A-rich foods of either animal or vegetable origin (<week^-1^) during the previous week in Brazzaville, 60% in Kouilou, and 71% in the rural north area. The infrequent consumers of yellow fruits accounted for 64.0% in Brazzaville, 77% in Kouilou, and 75% in the rural north.

The mean rates of intake of vitamin A of animal origin in Brazzaville, Pointe-Noire, and the rural north were respectively 5.0, 4.8, and 2.7 days per week. The mean weighted total consumption of animal and plant sources of vitamin A in Brazzaville, Kouilou, and the rural north was equal to 4.9, 4.4, and 3.4 days per week respectively.

### Vitamin A status

#### Clinical markers

The values of the clinical indicators of vitamin A deficiency obtained were much higher than the threshold values (4). A history of nightblindness was reported among 2% of the children in Brazzaville, 5% in Kouilou, and 8% in the rural north. The ophthalmologic examination was conducted on 2,696 children. Seventeen percent of the individuals in the rural north had Bitot spots whereas this symptom was detected in 6% of the children living in Brazzaville and 2% of those living in Kouilou (Table 3).

The rates of distribution of the clinical signs of xerophthalmia were as follows: stage XN: 5.2%, stage X_1_B: 8%, and stage X_2_, X_3_A: 2.5%. The prevalence of active xerophthalmia (X_2_, X_3_A) was 2.5% in both urban areas and rural north.

The prevalence of trachoma was 53% in Brazzaville, 1.8% in Kouilou, and 50% in the rural north. The prevalence of trachoma in urban areas and the rural north combined accounted for 50%.

#### Histological markers

Fifty-three percent of the 1,162 children tested had abnormal ICT findings (Table 3). The prevalence of deficient ICT (69%) was significantly higher among children in Brazzaville than among those in the rural north and Kouilou where the figures were 45% and 42% (p<0.001) respectively.

#### Biochemical markers

Of 207 samples available for the MRDR tests on DBS, 158 were suitable for analysis because of storage problems or technical constraints. Overall, 30% of the 158 children had MRDR test results (≥0.06) (Table 3). The proportion of the MRDR tests reflecting critical status (i.e. ≥0.06) was significantly higher in the Brazzaville area, where it reached 41%, than in the Kouilou area, where it was 17% (p<0.001).

Low levels of serum retinol (<10 μg/dL) were observed in 29% of the children (7.4±1.9 μg/dL) in the Brazzaville area, 25% (7.2±1.5 μg/dL) in the Kouilou area, and 26.5% (6.45±2.35 μg/dL) in the rural north. Low levels of serum retinol were observed in 27% (7.02±2.0 μg/dL) of all the individuals tested. Serum retinol concentrations ranging from 10 to 20 μg/dL accounted for 47% of the sample in both Brazzaville area (14.05±2.85 μg/dL) and rural north (14.55±2.65 μg/dL) and for 56% in the Kouilou area (14.0±3.0 μg/dL). However, 50% (14.2±2.83 μg/dL) of the individuals studied had serum retinol concentrations of 10-20 μg/dL.

A linear correlation was found to exist between morbidity and abnormal MRDR findings (p<0.001) (Table 4).

**Table 4.** MRDR test versus morbidity among Congolese preschool children in the regions studied

Region	Morbidity (diarrhoea)	
	MRDR ≥0.06* No. (%), 95% CI	MRDR <0.06* No. (%), 95% CI
Brazzaville (urban)	46/67 (69.0), 59.0-79.0	21/67 (31.0), 21.0-41.0
Kouilou (urban)	10/19 (53.0), 42.3-63.7	9/19 (47.0), 36.3-57.7
Rural north	ND	ND
All samples	56/86 (65.0), 54.8-75.2	30/86 (35.0), 24.8-45.2

χ^2^,

*p<0.001;

CI=Confidence interval;

MRDR=Modified relative-dose response;

ND=Not defined

#### Correlations between ICT tests and MRDR tests

A significant correlation was also found to exist between the ICT test and the MRDR findings (p<0.001, χ^2^=98.74) in the subsample (n=31) studied (Table 5).

**Table 5.** Rate of occurrence of vitamin A deficiency-related symptoms detected in various tests (ICT and MRDR) in preschool children inhabiting urban and rural Congolese regions

Test ICT*	Brazzaville (urban) No. (%), 95% CI	Kouilou (urban) No. (%), 95% CI	Rural north No. (%), 95% CI	All samples No. (%), 95% CI
N	3/31 (10.0), 0.0-20.7	27/43 (63.0), 48.3-77.7	6/14 (43.0), 16.6-69.4	36/88 (41.0), 40.0-42.0
M+	1/31 (3.0), 0.0-9.0	0	0	1/88 (1.0), 97.8-100
M-	0	0	1/14 (7.0), 0.0-21.0	1/88 (1.0), 0.98-1.0
D	27/31 (55.0), 37.1-72.9	16/43 (37.0), 22.3-51.7	7/14 (50.0), 23.3-76.7	50/88 (56.8), 56.6-57.0
MRDR*				
≥0.06	17/31 (55.0), 37.1-72.9	5/43 (12.0), 4.5-19.5	ND	22/74 (30.0), 19.4-40.6
<0.06	14/31(45.0), 27.0-63.0	38/43 (88.0), 80.5-95.5	ND	52/74 (70.0), 59.4-80.6

χ^2^=98.74,

*p<0.001;

CI=Confidence interval;

ICT=Impression cytology with transfer;

M+=Marginal+;

M-=Marginal-;

N=Normal;

D=Deficient;

ND=Not defined

## DISCUSSION

The results of the present survey extend our knowledge about the occurrence of xerophthalmia in the setting of the Republic of Congo. To the best of our knowledge, this is the first time vitamin A status has been assessed in preschool children in an equatorial African setting by performing the MRDR test on the DBS samples and ICT.

The MRDR test is an accurate indicator of vitamin A status of individuals. The effects of confounding factors, such as infectious diseases, on serum retinol concentrations are thought to be minimized, whether MRDR is performed on DBS or on fresh specimens (9). The MRDR test requires only one blood sample, and the samples have to be frozen and stored for subsequent analysis. Based on the DR/R ratio obtained on a single sample, the effects of storage on vitamin A stability and those of the sampling efficiency can also be predicted and minimized. Another advantage is that children can be tested first in the morning at their homes and brought back to the survey site a few hours later to complete the tests.

On the other hand, the MRDR test involves several constraints. Free DR is not stable once it has been extracted from the serum. Extreme care, therefore, has to be taken during the analytical procedure to protect the extracts from light and to inject them as soon as possible into the HPLC system. Since DR-acetate is not currently available commercially, it has to be synthesized or isolated from fish liver-oil (9). In addition, several precautions have to be ensured to obtain their compliance and adhesion in some cultural contexts while collecting blood samples from children. In our study, 49 of the MRDR tests on DBS were not completed in the rural north due to the difficulties and logistic constraints involved in the transport and storage of DR in the field. However, in other study areas, the procedure was carried out fairly satisfactorily.

In this survey, in which the ICT was applied to preschool children, the simplicity of the non-invasive method was confirmed and the smears obtained were quickly and easily interpreted. When the ICT data indicated the presence of deficient vitamin A status, the MRDR test was also consistently abnormal. An MRDR DR/R ratio of ≥0.06 is indicative of vitamin A deficiency (9). The results obtained here are very similar to those published in previous surveys (11). The ICT results of our study indicate the presence of a subclinical level of vitamin A deficiency whereas the MRDR tests showed values reflecting greatly depleted hepatic vitamin A reserves. In fact, there is no contradiction here as these two tests assess different aspects of vitamin A status. The ICT test reflects the effects, with some inertia, of a vitamin A deficiency in the epithelial tissue whereas the MRDR test indicates the current state of the liver stores (9). This difference would help explain why a close correlation was not consistently found to exist between the biochemical and the histological indicators. Similar results were previously obtained in surveys conducted in other African settings. Coutsoudis *et al*., for example, using the same approach and tools in Durban (South Africa), reported that 18% of ICT tests were abnormal and that 44.0% of the population tested had low retinol values (12). In the present survey, the handling procedures were rigorously conducted, and since the laboratory procedures were continuously checked, the critical values reported here, which are consistent with the WHO alarm thresholds, are certainly accurate.

The nutritional and health-related parameters, such as socioeconomic indicators, vitamin A intake, and co-morbidities, have to be considered to identify the factors responsible for vitamin A deficiency. The results of the present study showed that all the parameters studied were linked to both MRDR and ICT test results. The HKI food-frequency values (6) were very low in the whole population, aged less than six years, recruited in the Congolese areas studied. Vitamin A status was assumed to be at its lowest level during the study period: during the dry season, there is a shortage of vitamin A-rich fruits (mangoes) and vegetables, which no doubt explains the high prevalence of vitamin A deficits, and this factor may also account for the increase in child mortality which occurs between June and September. The present MRDR measurements showed the existence of abnormal or critical vitamin A status among nearly 30% of the Congolese children tested. This picture of vitamin A deficiency is due to the low intake of vitamin A and accounts for the high prevalence of clinical ocular symptoms, which are liable in addition to develop (or worsen) into active trachoma. In this study, the occurrence of serum retinol values of <10 μg/dL is consistent with greatly-depleted vitamin A reserves, since concentrations of the <20 μg/dL threshold reflect a low status, corresponding to a subclinical level of vitamin A deficiency. These findings are even more alarming than those published in previous studies on the same regions (13). Likewise, the prevalence of nightblindness and Bitot spots recorded was much higher than the respective WHO thresholds taken to define a public-health issue (4). The increase in the prevalence of vitamin A deficiency and trachoma is attributable to the greatly-degraded socioeconomic conditions pertaining in this country as the result of underdevelopment and the civil war which occurred in 1997 (14). The prevalence of trachoma was very high in the rural north (96%) compared to Kouilou (1.8%), where the vitamin A supplementation coverage was the lowest, probably because of the relatively-good socioeconomic conditions existing in this region at the time of the survey. In addition, the prevalence of active xerophthalmia at the national level was much higher than the WHO threshold of 0.01% taken to be an alert to the existence of a public-health issue (4).

The data obtained here should be interpreted taking into account the additional impact of the dry season when the study was carried out. It has been established that infectious diseases can precipitate the occurrence of xerophthalmia in children with low hepatic vitamin A reserves (15). Conversely, this condition might account for the prevalence of vitamin A deficiency in Congolese children. Diarrhoea is also a known risk factor contributing to vitamin A deficiency (15), and measles is responsible for increasing the tissular and peripheral requirements for this nutrient (16). In view of all these considerations, although we are aware of the possibility that some recall bias can affect the accuracy of reported events, we are confident that our findings are accurate because they are perfectly consistent with the data previously published (15).

To fight vitamin A deficiency, vitamin A supplementation campaigns constitute one of the best short-term strategies as previously described (3). It is, therefore, crucial to explore other approaches and to implement multiple strategies to reach all the target populations. It is also essential that all the strategies adopted should be reported and monitored to prevent the over-prescription of vitamin A.

### Conclusions

The results of the present study showed that vitamin A deficiency is still a serious public-health issue in the Congolese areas studied. The values of the clinical indicators to vitamin A deficiency obtained in our study were much higher than the threshold values established by the WHO. Our data showed that more than three-quarters of the study children had abnormal retinol stores. Fighting vitamin A deficiency is one of the priorities adopted by UNICEF Congo, the NGO MEDECINS D'AFRIQUE Brazzaville, and the Congolese Ministry of Public Health in 2010. Solving the problems associated with this and other forms of malnutrition in the long term will require improving the overall economic and social conditions under which a large proportion of the populations of all the countries involved are still living. In the medium term, the most promising strategies focus on increasing the vitamin A supply by improving the diet and the diversity of food supply, which seems to be the most thorough and durable method, enriching some foodstuffs with vitamin A and periodically administering strong doses of vitamin A. These methods can be used either separately or together. To prevent the occurrence of subclinical vitamin A levels and reach the most needy populations, we will have to reinforce the present strategies as follows: (a) by measuring the impact of distribution of vitamin A capsules in remote areas, using accurate indicators, and identifying the factors responsible for poor distribution and (b) by training health workers at integrated health centres and district hospitals in diagnostic techniques and methods of assessing vitamin A deficiency in preschool children and lactating and childbearing women.

Based on our experience, the ICT test can be taken to be a good and reliable indicator of peripheral vitamin A deficiency. We suggest that this test should be used in vitamin A deficiency-screening programmes in developing countries because it is less expensive and more convenient than the other available methods. The MRDR test on DBS is also an accurate means of determining vitamin A status. The simplicity of this method and the fact that it can be easily applied in the field make it a good tool for conducting assessments of vitamin A status in sub-Saharan African settings.

## ACKNOWLEDGEMENTS

This study was financed by UNICEF Brazzaville. The authors thank the team at the NGO MEDECINS D'AFRIQUE Brazzaville and the Congolese Ministry of Research and Education for their assistance. They also thank Mrs. Régine Luzeau for her practical advice and Mrs. Evelyne Makanga for her helpful collaboration.
